# METH-Induced Neurotoxicity Is Alleviated by Lactulose Pretreatment Through Suppressing Oxidative Stress and Neuroinflammation in Rat Striatum

**DOI:** 10.3389/fnins.2018.00802

**Published:** 2018-11-02

**Authors:** Xiao-Li Xie, Wen-Tao Zhou, Kai-Kai Zhang, Li-Jian Chen, Qi Wang

**Affiliations:** ^1^Department of Toxicology, School of Public Health, Southern Medical University (Guangdong Provincial Key Laboratory of Tropical Disease Research), Guangzhou, China; ^2^Department of Forensic Pathology, School of Forensic Medicine, Southern Medical University, Guangzhou, China

**Keywords:** METH, neuroinflammation, oxidative stress, lactulose, Nrf2/HO-1 axis

## Abstract

Abuse of methamphetamine (METH) results in neurological and psychiatric abnormalities. Lactulose is a poorly absorbed derivative of lactose and can effectively alleviate METH-induced neurotoxicity in rats. The present study was designed to investigate the effects of lactulose on METH-induced neurotoxicity. Rats received METH (15 mg/kg, 8 intraperitoneal injections, 12-h interval) or saline and received lactulose (5.3 g/kg, oral gavage, 12-h interval) or vehicle 2 days prior to the METH administration. Reactive oxygen species (ROS) and malondialdehyde (MDA) were measured. Protein levels of toll-like receptor 4 (TLR4), myeloid differentiation factor 88 (MyD88), tumor necrosis factor receptor associated factor 6 (TRAF6), nuclear factor κB (NFκB), interleukin (IL)-1β, IL-6, TNF-α, cleaved caspase 3, and poly(ADP-ribose) polymerase-1 (PARP-1) were determined by western blotting. mRNA expressions of nuclear factor erythroid 2-relatted factor-2 (Nrf2), p62, and heme oxygenase-1 (HO-1) were assessed by RT-qPCR. The lactulose pretreatment decreased METH-induced cytoplasmic damage in rat livers according to histopathological observation. Compared to the control group, overproduction of ROS and MDA were observed in rat striatums in the METH alone-treated group, while the lactulose pretreatment significantly attenuated the METH-induced up-regulation of oxidative stress. The lactulose pretreatment significantly repressed over-expressions of proteins of TLR4, MyD88, TRAF6, NFκB, IL-1β, IL-6, TNF-α, cleaved caspase 3, PARP-1. The lactulose pretreatment increased mRNA expressions of Nrf2, p62, and HO-1. These findings suggest that lactulose pretreatment can alleviate METH-induced neurotoxicity through suppressing neuroinflammation and oxidative stress, which might be attributed to the activation of the Nrf2/HO-1 axis.

## Introduction

Methamphetamine (METH) is a popular new-type psychostimulant drug that may result in neurotoxicity. METH-induced neurotoxicity may be related to apoptosis (Jumnongprakhon et al., 2014), oxidative stress (Nguyen et al., 2015; Wen et al., 2016) and inflammatory changes (Gonçalves et al., 2010; Park et al., 2017). Overproduction of reactive oxygen species (ROS) induced by METH may play a key role in oxidative damage (Gluck et al., 2001). METH can also trigger a neuroinflammatory process by releasing pro-inflammatory molecules, acting as processors (Coelho-Santos et al., 2012; Park et al., 2017). The pro-inflammatory molecules may indirectly result in neurotoxicity and the activation of glial cells, which could exacerbate neuroinflammation (Park et al., 2017).

Nuclear factor erythroid 2-relatted factor-2 (Nrf2) is a fundamental regulator of antioxidant response element-dependent transcription. It plays a significant role in the cellular adaptive response to oxidative stress (Yang et al., 2018). Besides its antioxidant function, Nrf2 activation also plays a central role in the regulation of inflammation (Kuhn et al., 2011). Under unstressed conditions, a low level of Nrf2 is maintained by Kelch-like ECH-associated protein 1, while under oxidative stress conditions, Nrf2 is released to activate antioxidant response elements (e.g., heme oxygenase-1, HO-1) in the nucleus (Suzuki et al., 2013). Sequestosome-1 (SQSTM1, p62) expression can prevent Nrf2 degradation and enhance its nuclear accumulation (Sun et al., 2016). In addition, p62 is a target gene of Nrf2 (Jain et al., 2010) and they can form a positive feedback loop by inducing an antioxidant response element and an anti-inflammatory effect.

Lactulose is a non-digestible galactose-fructose disaccharide. Lactulose is metabolized in the colon by bacterial flora to short-chain fatty acids, which increases H^+^ concentration and promotes the formation of ${\text{NH}}_{4}^{+}$ from NH_3_ (ammonia) in the colon. Accumulation of ammonia in the colon effectively reduces serum ammonia concentration and subsequently alleviates adverse effects of hyperammonemia (Moratalla et al., 2017), such as neurotoxicity, neurocognitive defects. Therefore, lactulose can be used as prevention and treatment of hepatic encephalopathy with cirrhosis, as it can effectively improve patients' neurocognitive impairment and reverse low-grade cerebral edema by preventing hyperammonemia and inflammation (Rai et al., 2015; Moratalla et al., 2017). In this study, rats were pretreated with lactulose/vehicle and administered with METH/saline. Focusing on oxidative stress, inflammatory responses and the Nrf2/HO-1 axis, the effects of lactulose on METH-induced neurotoxicity in rat striatum were clarified.

## Materials and methods

### Chemicals

METH (purity of 99.1%, identified by the National Institute for Food and Drug Control, Guangzhou, China) was purchased from the National Institute for the Control of Pharmaceutical and Biological Products (Beijing, China). Lactulose was obtained from Pharmaceutical Associates Inc., Greenville, SC. DCFH-DA was purchased from Sigma Chemical Co (St. Louis, MO, USA).

### Animals and treatments

A total of eighteen male Sprague Dawley rats (5-weeks-old) were purchased from the Laboratory Animal Center of Southern Medical University (Guangzhou, China). The rats were singly housed in plastic cages in an animal facility maintained under standard conditions (room temperature, 23 ± 1°C; relative humidity, 44 ± 5%; and a light/dark cycle of 12 h) and given free access to a basal diet and water. The animals were acclimatized for 1 week prior to the beginning of the experiment. This study was reviewed and approved by the National Institutes of Health Guide for the Care and Use of Laboratory Animals of the Southern Medical University.

Briefly, the rats were randomly divided into 3 groups (6 rats in each group). The rats received 8 intraperitoneal (i.p.) injections of METH (15 mg/ml/kg body weight/injection) or saline (1 ml/kg) at 12 h (h) intervals. When exposed to this dose, rats have a similar concentration of METH in the blood at 1 h after the last injection to the median value of METH in the blood of METH abusers (Melega et al., 2007; Huang et al., 2015). Therefore, the single dose of METH was chosen based on previous studies (Huang et al., 2015; Wang et al., 2017). Two days prior to the METH treatment, the rats were pretreated with lactulose (5.3 g/kg body weight, oral gavage, every 12 h) or vehicle (100 mg/mL galactose and 80 mg/mL lactose) until the day before sacrifice. The dose of lactulose, which was chosen in this study, could effectively enhance ammonia excretion and has been used as an treatment for the cirrhosis patients with hepatic encephalopathy and neurocognitive defects (Jia and Zhang, 2005; Nicaise et al., 2008; Al Sibae and McGuire, 2009; Northrop et al., 2016). All rats were killed by rapid decapitation 24 h after the last injection of METH/saline. The livers as well as the striatums were quickly excised. The livers were fixed in 10% phosphate-buffered formalin for histopathological observation and the striatums were stored at −80°C for subsequent analyses.

### Histopathological observation

Liver tissues were embedded in paraffin, sectioned at 3-μm thickness, and stained with hematoxylin and eosin (H&E) for histopathological examination.

### Detections of ROS production in rat striatum

Striatum tissues were washed with ice-cold PBS. Then they were made into single-cell suspension by homogenizer and centrifuged at 500 g for 10 min at 4°C. After being washed twice with ice-cold PBS, the cells were re-suspended. The re-suspension solution was divided into two parts: One part was used to determine the protein content after ultrasonic disruption using the Bradford protein assay kit (Bio-Rad, Hercules, CA), and the other part was incubated with 10 μM DCFH-DA (Sigma), kept out of light for 30 min at 37°C and washed twice with ice-cold PBS. DCFH-DA fluorescence was determined by flow cytometry (BD LSRFortessa^TM^, BD, CA, USA). The results of ROS generation were calculated as DCFH-DA fluorescence per microgram and expressed as fold changes compared with the mean value of the control group.

### Measurement of malondialdehyde (MDA) content in rat striatum

Striatums of rats were homogenized in RIPA lysis buffer on ice and centrifuged at 12,000 g for 10 min at 4°C to collect the supernatant. The total protein content was tested with the Bradford protein assay kit (Bio-Rad, Hercules, CA). MDA content was determined using a Lipid Peroxidation MDA Assay Kit (Nanjing Jiancheng Bioengineering Institute, China) following the manufacturer's instructions. MDA content was calculated and expressed as nanomole per microgram (nmol/mg) protein.

### Real-time quantitative RT-PCR (RT-qPCR) analysis for Nrf2/HO-1 axis in rat striatum

Briefly, cDNA copies of total RNA were obtained using a PrimeScript™ RT Master Mix (RR036A, Takara Biotechnology Co., LTD.). RT-qPCR was conducted using Premix Ex TaqTM GC (RR820A, Takara) on the StrataGene MX 3005P Multiplex Quantitative PCR System (Agilent Technologies, USA), with primers for Nrf2, HO-1, and p62. The PCR program cycles were set as follows: initial denaturing at 95°C for 30 s, followed by 40 cycles at 95°C for 15 s, and 60°C for 30 s. β-actin was used as an internal standard, and the mRNA levels of the target genes were normalized to β-actin. mRNA expressions in the METH alone and the METH plus lactulose groups were displayed as fold changes compared to the mean value of the control group. All the RT-qPCR experiments were performed in triplicate. Detailed information of the primers is listed in Supplementary Table 1.

### Western blotting analysis for inflammatory-related factors and apoptosis in rat striatum

Proteins were extracted from the rat striatum as described previously (Wang et al., 2017). The proteins were separated by SDS-polyacrylamide gel electrophoresis (PAGE). After electrophoresis, the proteins were transferred to polyvinylidine difluoride membranes. The membranes were then blocked with 5% milk-Tris-buffered solution-Tween solution for 2 h and subsequently incubated overnight at 4°C followed by appropriate secondary antibodies for 2 h at room temperature. Bands were visualized using the ECL system (BIO-RAD Laboratories, Inc., California, USA). Primary antibodies against toll-like receptor 4 (TLR4, sc-293072, Santa Cruz Biotechnology), myeloid differentiation factor 88 (MyD88, sc-74532, Santa Cruz), tumor necrosis factor (TNF) receptor associated factor 6 (TRAF6, sc-8409, Santa Cruz), nuclear factor (NF) κB (sc-8008, Santa Cruz), interleukin (IL)-1β (sc-12742, Santa Cruz), IL-6 (sc-57315, Santa Cruz), TNF-α (sc-12744, Santa Cruz), caspase 3 (9665, Cell Signaling Technology), poly(ADP-ribose) polymerase-1 (PARP-1, sc-8007, Santa Cruz), and GAPDH (sc-32233, Santa Cruz) were used.

Densitometric analysis was conducted using Tanon Gel Image System (version 4.2). Data of relative integrated optical density values of bands are presented as bar charts.

### Statistical analysis

All values were expressed as means ± SEM. Statistical analyses were conducted using the scientific statistics software SPSS (version 16). One-way analysis of variance (ANOVA) with repeated measures, followed by *post-hoc* Tukey tests, was used for comparisons of multiple groups. Values of *p* < 0.05 were considered as statistically significant.

## Results

### Lactulose decreased METH-induced hepatotoxicity in rats

**Figure 1:**
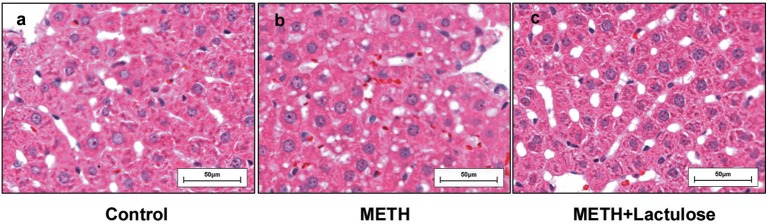
Histopathological observation of rat livers. **(a)** No pathological change was observed in the control group. **(b)** Vacuolar degeneration in the cytoplasmic was observed in the METH alone-treated rats. **(c)** The cytoplasmic damage was obviously attenuated by the pretreatment with lactulose.

Extensive cytoplasmic damage was observed in the livers of the METH alone-treated rats (Figure 1b), while no obvious changes of hepatocellular morphology were observed in the control group (Figure 1a) by histopathological observation. METH-induced changes in hepatocellular morphology were attenuated under pretreatment with lactulose (Figure 1c).

### Lactulose suppressed overproductions of ROS and MDA induced by METH in rat striatum

**Figure 2:**
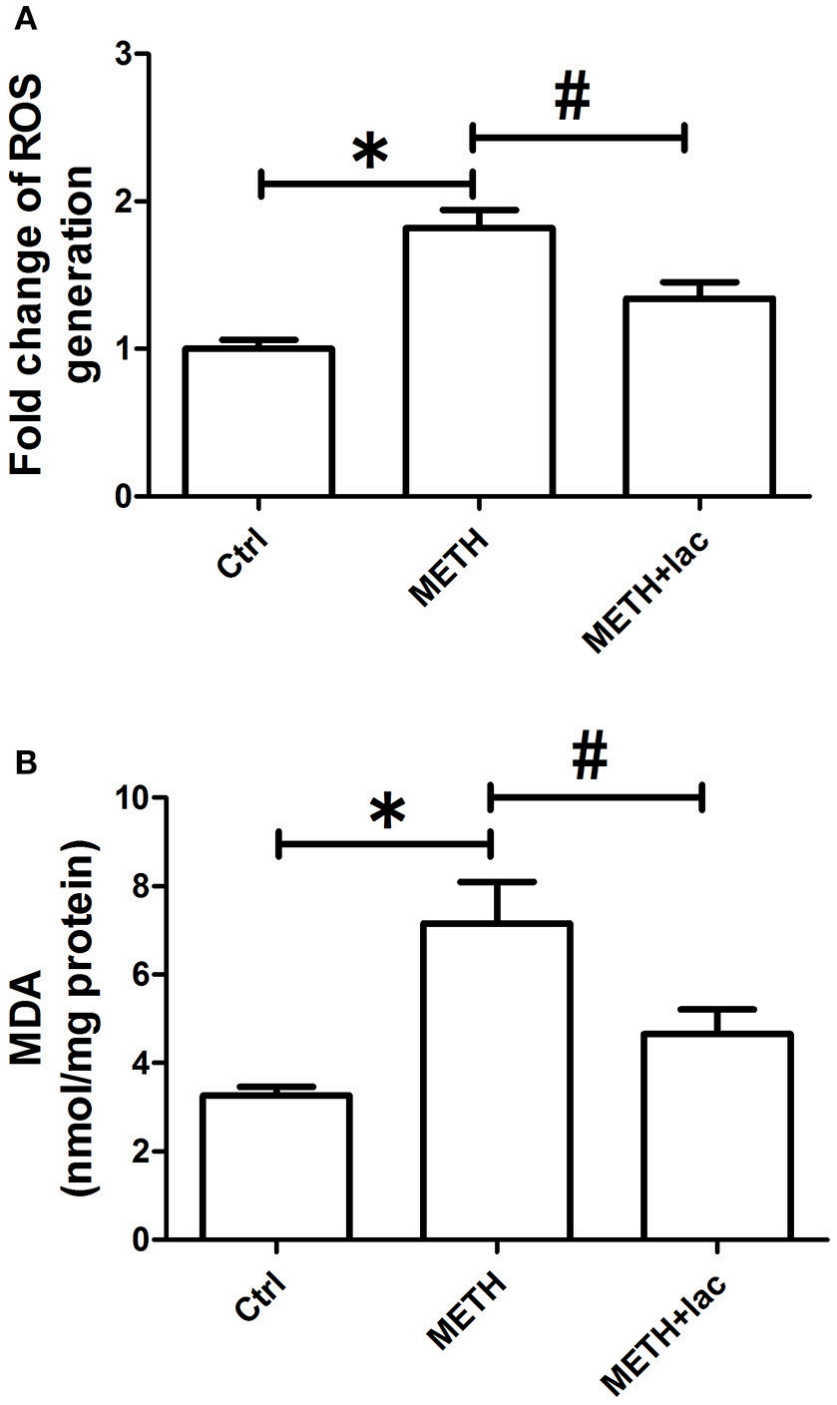
Detection of ROS generation **(A)** and MDA production **(B)** in rat striatum. **(A)** Compared to the control group, over-generation of ROS was detected in the METH alone-treated rats, while the pretreatment with lactulose significantly decreased the ROS generation induced by METH. Significant compared to the control group (*p < 0.05); Significant compared to the METH alone-treated group (#*p* < 0.05). Lact, Lactulose. **(B)** Overproduction of MDA was observed in the METH alone-treated rats compared to the control group. The pretreatment with lactulose significantly suppressed the production of MDA resulting from the METH treatment. Significant compared to the control group (*p < 0.05); Significant compared to the METH alone-treated group (#*p* < 0.05). Lact, Lactulose.

As shown in Figure 2A, the ROS level was more significantly augmented in the METH alone-treated rats than that in control rats, which indicates the pro-oxidative effect of METH. However, the pretreatment with lactulose markedly decreased METH-induced increasing of ROS compared with the METH alone-treated group. Consistently, Figure 2B shows that the MDA content significantly increased in the METH alone-treated group compared with the control group, while the pretreatment with lactulose effectively suppressed the increase.

### Lactulose up-regulated mRNA expressions of Nrf2/Ho-1 axis, decreased protein expressions of inflammatory-related factors and suppressed apoptosis in rat striatum

**Figure 3:**
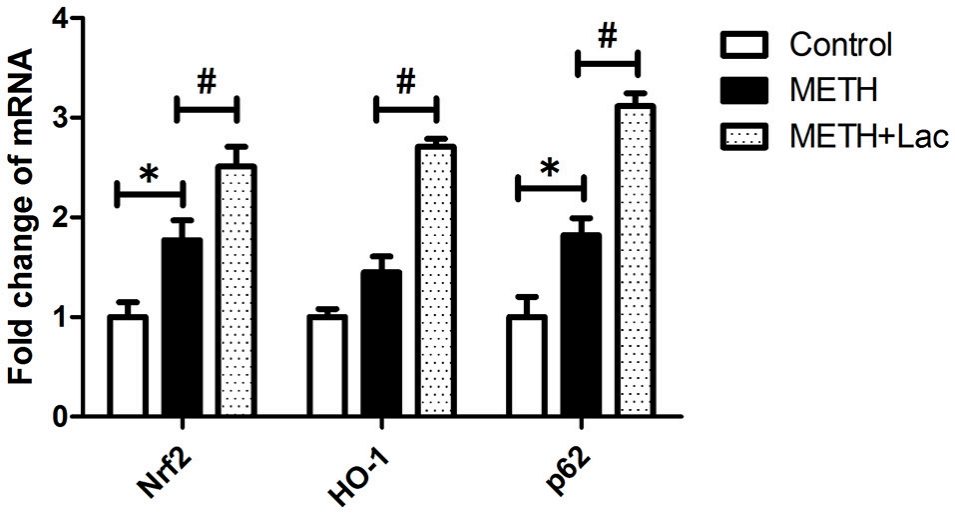
RT-qPCR analysis for genes of *Nrf2, HO-1*, and *p62* in rat striatum. Compared to the control group, mRNA expressions of Nrf2 and p62 were significantly up-regulated in the METH alone-treated rats, while no change of HO-1 was observed. The pretreatment with lactulose significantly increased mRNA expressions of Nrf2, HO-1 and p62 compared to the METH alone-treated rats. Significant compared to the control group (*p < 0.05); Significant compared to the METH alone-treated group (#*p* < 0.05). Lact, Lactulose.

**Figure 4:**
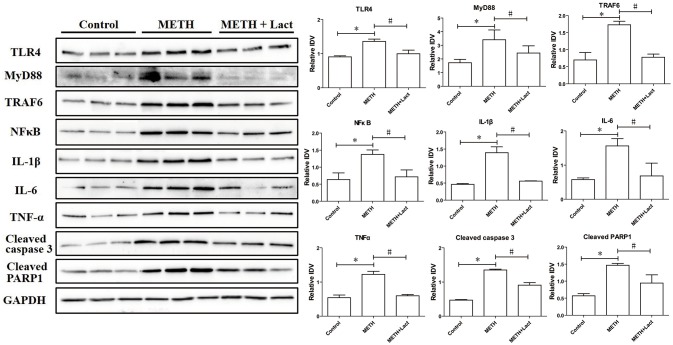
Western blotting analysis for protein expressions of TLR4, MyD88, TRAF6, NFκB, IL-1β, IL-6, TNF-α, cleaved caspase 3 and PARP1 in rat striatum. Compared to the control group, expressions of TLR4, MyD88, TRAF6, NFκB, IL-1β, IL-6, TNF-α, cleaved caspase 3 and PARP1 significantly increased in the METH alone-treated group, while the lactulose pretreatment effectively alleviated these over-expressions, suggesting suppressions of neuroinflammation and apoptosis induced by METH. Significant compared to the control group (*p < 0.05); Significant compared to the METH alone-treated group (#*p* < 0.05). Lact, Lactulose.

The lactulose pretreatment markedly up-regulated mRNA expressions of Nrf2, HO-1, and p62, compared with the METH alone-treated group (Figure 3). The lactulose pretreatment significantly repressed over-expressions of proteins of TLR4, MyD88, TRAF6, NFκB, IL-1β, IL-6, and TNF-α induced by METH. Expressions of cleaved caspase 3 and PARP1 were substantially decreased by the lactulose pretreatment compared to the METH-alone group (Figure 4).

## Discussion

In this study, obvious hepatic injury can be detected after METH alone treatment, showing as cytoplasmic vacuolar degeneration. However, the lactulose pretreatment effectively reduced the cytoplasmic damage. These findings indicate that the model was successfully established (Halpin and Yamamoto, 2012).

Overproduction of ROS was observed in striatums of the METH alone-treated group, indicating the pro-oxidative effects of METH. Overproduction of MDA, a final product of lipid peroxidation, was found in striatums of the METH alone-treated group, suggesting the induction of oxidative stress. However, when compared with the METH alone-treated rats, the lactulose pretreatment significantly attenuated productions of ROS and MDA. These findings indicated that pretreatment with lactulose can suppress METH-induced up-regulation of oxidative stress.

Toll-like receptors (TLRs) are a class of immunological pattern recognition receptors that play a fundamental role in pathogen recognition as well as in inflammatory responses. TLR4, also called CD284, can recruit the adaptor proteins (for example MyD88) bind to TRAF6, and then trigger NFκB activation to induce the transcription of pro-inflammatory cytokines, such as IL-6, IL-1β, TNF-α (Moscat et al., 2006; Zhang et al., 2013; Wang et al., 2018). Previous studies have reported that TLR4 plays an important part in METH-induced neuroinflammation (Du et al., 2017). In this study, over-expressions of proteins of TLR4, MyD88, and TRAF6 were observed in the METH alone-treated rats when compared with the control group. Consistently, protein expressions of NFκB as well as the pro-inflammatory cytokines, IL-6, IL-1β, and TNF-α, were also significantly up-regulated after METH treatment. These results confirmed that METH treatment induced neuroinflammation by activating the TLR4/NFκB pathway, whereas decreased protein expressions of TLR4, MyD88, TRAF6, NFκB, IL-6, IL-1β, and TNF-α were observed in the lactulose-pretreated group, suggesting the alleviation of neuroinflammation by lactulose.

The Nrf2/HO-1 axis is commonly referred to as an antioxidant system, which can be activated by ROS overproduction (Suzuki and Yamamoto, 2017). Besides its anti-oxidative function, Nrf2/HO-1 axis is an important part of the regulation of inflammation (Kuhn et al., 2011). In our previous study, in the whole-cell lysates of rat striatum, METH-induced over-expression of Nrf2 and p62 protein leves were significantly attenuated by the lactulose pretreatment. However, in cell nucleus, protein expressions of Nrf2 and HO-1 obviously decreased in METH alone-treated rats, but increased by the pretreatment with lactulose compared to the METH alone-treated rats, suggesting excessive accumulation of Nrf2 in cytoplasm paradoxically repressed Nrf2 nuclear transformation and induction of HO-1 from the level of protein (Xie et al., 2018). In this study, mRNA expressions of Nrf2, HO-1 and p62 in the whole-cell lysates of rat striatum were examined. Compared with the control group, the mRNA expression of Nrf2 was markedly up-regulated by METH treatment, whereas no significant change of HO-1 in the mRNA level was observed. These findings might suggest that as one of the downstream response elements of Nrf2, HO-1 was not effectively activated in rat striatums at transcription level. Furthermore, mRNA expression of p62 was significantly up-regulated in the striatums of METH alone-treated rats, while the lactulose pretreatment further increased mRNA expressions of Nrf2 and its targets, HO-1 and p62, suggesting activation of Nrf2/HO-1 axis by lactulose. The lactulose pretreatment can induce nucleus translocation of Nrf2. Therefore, to maintain the activation of Nrf2/HO-1 axis, mRNA expression of Nrf2 were further up-regulated by the lactulose pretreatment. Moreover, p62 is a target gene for Nrf2 and can create a positive feedback loop by inducing a downstream response element (Jain et al., 2010). In addition, p62 can be selective turnover by autophagy. Lactulose pretreatment increased turnover of p62 by alleviating impaired autophagy flux and decreased p62 protein expression (Xie et al., 2018). Thus, in the lactulose pretreatment group, the increased mRNA levels and decreased protein expressions of Nrf2 and p62 were observed in the whole-cell lysates of rat striatum when compared with METH-alone group, which may be caused by differences in translation efficiency or RNA/protein kinetics, though further investigations are needed to clarify the underlying mechanism.

Compared with the control group, increased expressions of proteins of cleaved caspase 3 and PARP1 were found in the METH alone-treated rats, implying its neurotoxicity. Previous studies have reported that METH-induced increase of oxidative stress and pro-inflammatory cytokines may activate downstream apoptosis (Allagnat et al., 2012; Park et al., 2017), which may play an important role in METH-induced neurotoxicity. However, in this study, decreased protein expressions of cleaved caspase 3 and PARP1 were observed in lactulose-pretreated rats, suggesting alleviation of METH-induced neurotoxicity.

METH abuse could also induce obvious neurocognitive defects (Cuzen et al., 2015), which is correlated with high serum ammonia levels. Lactulose could improve neurocognitive scores by reducing serum ammonia in cirrhotic patients with minimal hepatic encephalophthy (Moratalla et al., 2017). Therefore, we speculated that the treatment with lactulose might attenuate METH-induced neurotoxicity as well as neurocognitive defects and be favorable to improving the therapeutic effects of METH intoxication/addiction, at least at certain exert, which should be confirmed in further clinical practices.

In summary, oxidative stress and neuroinflammation induced by METH may play an important role in its neurotoxicity, while pretreatment with lactulose can alleviate the neurotoxicity through repressing oxidative stress and decreasing neuroinflammation, which might attribute to the activation of Nrf2/HO-1 axis.

## Author contributions

X-LX drafted the manuscript and carried out WB/RT-qPCR experiments. W-TZ carried out animal experiment. K-KZ and L-JC carried out WB/ RT-qPCR experiments. QW designed the study. All authors read and approved the final manuscript.

### Conflict of interest statement

The authors declare that the research was conducted in the absence of any commercial or financial relationships that could be construed as a potential conflict of interest.
