# Supplementary material for: METH-Induced Neurotoxicity Is Alleviated by Lactulose Pretreatment Through Suppressing Oxidative Stress and Neuroinflammation in Rat Striatum
Source: Front Neurosci. 2018 Nov 2;12:802. doi: 10.3389/fnins.2018.00802 (PMC6224488; doi:10.3389/fnins.2018.00802)
Supplement: Supplementary file 1 [file Table_1.docx]

Supplemental Table 1. Primers for RT-qPCR analysis

| Gene | F | R |
| --- | --- | --- |
| Nrf2 | 5’-ATTGCTGTCCATCTCTGTCAG-3’ | 5’-GCTATTTTCCATTCCCGAGTTAC-3’ |
| HO-1 | 5’-GCCTGGCACATTTCCCTCAC-3’ | 5’-CAGAACAGCCGCCTCTACCG-3’ |
| p62 | 5’-AGAATGTGGGGGAGAGCGTGGC-3’ | 5’-GGGTGTCAGGCGGCTTCTCTT-3’ |
| β-actin | 5’-CGTGAAAAGATGACCCAGATCA-3’ | 5’-AGAGGCATACAGGGACAACACA-3’ |
